# “Choice Set” for health behavior in choice-constrained settings to frame research and inform policy: examples of food consumption, obesity and food security

**DOI:** 10.1186/s12939-016-0336-6

**Published:** 2016-03-16

**Authors:** Robert V. H. Dover, Estelle V. Lambert

**Affiliations:** Departamento de Antropología, Universidad de Antioquia, Medellin, Colombia; Division of Exercise Science and Sports Medicine, Department of Human Biology, Faculty of Health Sciences, University of Cape Town, PO Box 115, Newlands, 7725 Cape Town, South Africa

## Abstract

**Objectives:**

Using the nexus between food consumption, food security and obesity, this paper addresses the complexity of health behavior decision-making moments that reflect relational social dynamics in context-specific dialogues, often in choice-constrained conditions.

**Methods:**

A pragmatic review of literature regarding social determinants of health in relation to food consumption, food security and obesity was used to advance this theoretical model.

**Results and discussion:**

We suggest that health choice, such as food consumption, is based on more than the capacity and volition of individuals to make “healthy” choices, but is dialogic and adaptive. In terms of food consumption, there will always be choice-constrained conditions, along a continuum representing factors over which the individual has little or no control, to those for which they have greater agency. These range from food store geographies and inventories and food availability, logistical considerations such as transportation, food distribution, the structure of equity in food systems, state and non-government food and nutrition programs, to factors where the individual exercises a greater degree of autonomy, such as sociocultural foodways, family and neighborhood shopping strategies, and personal and family food preferences. At any given food decision-making moment, many factors of the continuum are present consciously or unconsciously when the individual makes a decision. These health behavior decision-making moments are mutable, whether from an individual perspective, or within a broader social or policy context. We review the construct of “choice set”, the confluence of factors that are temporally weighted by the differentiated and relationally-contextualized importance of certain factors over others in that moment. The choice transition represents an essential shift of the choice set based on the conscious and unconscious weighting of accumulated evidence, such that people can project certain outcomes. Policies and interventions should avoid dichotomies of “good and bad” food choices or health behaviors, but focus on those issues that contribute to the weightedness of factors influencing food choice behavior at a given decision-making moment and within a given choice set.

## Background

Eighty percent of all deaths due to non-communicable diseases (NCDs) occur in lower-and-middle-income countries (LMICs) [1]. These conditions are often referred to as chronic diseases of “lifestyle”, based on the underlying premise that they are caused by certain modifiable behaviors such as tobacco use, obesity and unhealthy eating, lack of physical activity, excessive intake of alcohol, and risky sexual behavior. Consistent with the epidemiological, nutrition and demographic transitions that characterize development [2], there is growing recognition that the social, environmental, political and economic bases of health and health–seeking behavior, such as poverty and the uneven distribution of wealth, lack of education, rapid urbanization and population ageing, have contributed to the rising prevalence of NCDs [3, 4]. Failure to recognize the complex and iterative nature of these influencers, at the individual, community, and policy levels, with respect to local context, will undermine efforts to prevent and manage the burden of disease [5]. In the words of Manderson and Whitehead [5] in their book on Global Health Policy: Local Reality and the Fallacy of the Level Playing Field, “the assumption of uniformity of context may be necessary to the process of planning global health programs, but also may create needless barriers to their effective execution”.

### Complexity of health decision-making moments

Global obesity indicators are not diminishing, in particular, in LMICs, despite international and national policy interventions. These secular trends have largely been attributed to the social determinants of health, “Big Food” and declining physical activity levels. However, in contrast, it is likely that the impact of local context and relative deprivation on obesity and health outcomes is not uniform, nor predictable, on the basis of the same set of variables in every community or household. Using examples concerning food consumption, food insecurity, and obesity, this paper addresses the methodological and conceptual complexities of individual health behavior decision-making moments, beyond a linear model or paradigm of undifferentiated causality.

We see these *moments* as relational social dynamics [6, 7] that engage different level actors, including the individual, in a context-specific dialogue about health behavior decisions, often in choice-constrained circumstances. In effect, the dialogic nature of such an ecological model suggests that individuals are both actors and agents in the construction of their health status. This dynamic is often over-looked in the development of individual- and community-based health interventions.

One example of this complexity concerning health decision-making moments may be illustrated by “the work-family interface” [8] in which employment is generally associated with better health status, while, by contrast, low-income earners may be burdened with longer work hours and little job security, resulting in a negative work-life spillover. Devine and colleagues [8] have explored this work-life spillover in relation to family food coping strategies. In their example, ‘serial eating’ (members of the family eating at different times) and the intake of highly processed foods are a trade-off between healthy eating, and convenience due to the scarcity of time or the use of food as a treat or reward. Moreover, the working mothers interviewed in this example expressed ambivalence and conflict in their maternal role, over not being able to prepare healthy meals for their children vs. ensuring that at least “they had some food in their bellies before going to bed”. These conscious trade-offs may perpetuate poor nutritional habits and associated negative health outcomes. Thus, the decision-making moments that impact on health status, are made within a more comprehensive context which may include pragmatic influences such as expediency and competing priorities, and more abstract vectors, based on individual value systems, or more complex values hierarchies.

Furthermore, these health behavior decision-making moments are mutable, whether from an individual perspective such as: disposable income, personal health belief systems, health identity and current health status, or within a broader social context, for example: community or cultural value system and social norms, or state-implemented programs and policies. Pescosolido sees it as a shift in “focus from individual choice to socially-constructed patterns of decisions, including consultation with others” [9].

### Meaningful contexts for health behavior choices: choice set and choice transition

We do not mean to say that health behavior choices are not about health, but they also concern social, and environmental, and policy determinants or personal and family circumstances, [10] and, more specifically, both the capacity and volition of the individual to make informed choices within their specific social context [11]. By *informed*, we refer to an awareness of the consequences of making a particular health behavior decision and the deliberateness of that decision. The convergence of factors that comprise what we refer to as the “choice set” for a specific decision are temporally weighted by the differentiated and relationally-contextualized importance of certain factors over others, in a given decision-making moment.

The moments are individual, but still occur in a larger social and experiential context where community, network and/or cohort evaluations of different variables offer a socially interpretive paradigm for individual health behavior decisions. These become structural and formative ideations [9], what Roseberry [12] calls “meaningful contexts”, that enter into the choice set as significant and weighted variables, particularly if they contribute to a working knowledge of or have an interpretive influence over health and health behavior decisions.

One such example of ‘meaningful contexts’ can be illustrated by a recent ruling against the New York City Health Board’s 2012 policy, limiting container sizes for the sale of sugar-sweetened beverages [13]. Although designed to address the public health issue of increasing prevalence of obesity and diabetes, the policy inadvertently mobilized external agents, food companies and business associations. These agents subsequently convinced the Afro-American community, the most affected and vulnerable, to contest the policy, by constructing an argument of racial targeting and infringement of rights and alleging an inequitable constraint on individual health behavior choice [14]. A seemingly, straightforward “meaningful context” of a local health policy, aimed at addressing environmental drivers of obesity, was altered by external actors, with vested interest, into a racially-polarised “meaningful context”. This example also illustrates that framing individual health behavior choice in a larger societal and policy context recognizes the dynamic relationship between micro-, meso- and macro- level actors and agents, and that the contexts in which people make health behavior decisions are constantly changing.

This dynamic relationship between different level actors also leads to surprising determinant variables. Estrade et.al. [15], in a study of food offerings from vendors located outside of lower income level schools in Scotland, show how meso-level fast food providers balance competing objectives: an awareness of the need to offer healthy fast foods to vulnerable students, a policy environment that looks with disfavor at the growing obesity problem, and a student clientele that bases their food choices on both traditional food ways and value for money. Fast food vendors feel they are forced to respond to their clientele to remain in business, suggesting that there is a great deal of protagonism at the micro-level of actors, and that the macro- and meso- level health messages compete with other, often incompatible, micro- and value-ladened variables such as tradition, economic viability and collaborative actors.

The community perspective on this nexus between food choice, food insecurity and obesity was recently explored with members of First Nation communities in Canada. In focus groups, participants were asked about factors that impacted on their ability to provide healthy and “culturally appropriate” food for their families [16]. Without prompting, the unaffordability of healthy food and associated complexities of low income, such as reliance on public transport or less frequent shopping trips and lack of access to or expiration of fresh foods, were highlighted as key factors. However, in some cases, the very solutions, such as food banks, were seen to exacerbate the problem. Participants spoke of the fact that the food was often of poor quality, close or past the expiration date, and they shared their experiences of the stigma and shame associated with using these outreach programs.

“It takes a lot to swallow your pride to access these resources, and if you’ re gonna go there and be judged by the person …that’s supposed to be helping you… we are a small community… and you walk through a door and your neighbors are sitting there at the table volunteering.”

And even when there was good quality food or fresh foods provided, there was a perception by some participants that community members may not know how to or may not have the facilities to prepare it.

These community perspectives can be contextualized against the concept of “vital places” put forward by Walton [17]. “Vital places” may be described as prominent, regularly frequented places within low-income (or in fact, any) neighborhoods, that relate to the health and well-being of communities, through social or behavioral mechanisms. In her study of the specific low-income, multi-ethnic community of Bayview, Wisconsin, one of the “vital places” that emerged, which crossed ethnic, gender and age boundaries, was a small, but comprehensive, affordable ethnic grocery within walking distance of most households.

This apparent contradiction to “food deserts” in low-income communities works because shop-owners “…know their market; it has risen in importance to the residents as a community fixture because it understands and responds to the community's needs and preferences.” One elderly resident explained, “I always go. It's close! It's not far and you can buy whatever you want … rice, peppers, meat. And then you come home. It's easy for Hmong people.”. By “easy” Walton [17] argues that this refers not only to geographic proximity, but that by offering more traditional, ethnic and culturally-appropriate foods, it also becomes more relationally-proximal. She suggests that researchers and policy makers should focus their interventions on existing assets and a “group-empowerment” agenda, for persons with limited social mobility and means. This “anomalous” example of a successful, low-income neighborhood, due in part, to the presence of “vital places” such as this grocery store, demonstrates the iterative nature of health behavior choices, social determinants and what can be achieved through involving community meso-level actors.

This micro- to meso-level relationship between the individual health behaviors and their social context is suggested by Pescosolido [9] where,

“a particular action, choice, or decision is embedded in a social process where the network interactions of individuals not only influence preference formation and define the situation but also drive the process of deciding whether something is wrong, whether anything can be done about it, what should be done, and how to evaluate the results.”

The significance of the idea of choice set lies in the ability to frame and articulate the relationship between factors, not as discrete entities that act upon the individual, but as the individual’s ability to invoke the social, environmental and policy context at the decision-making moment.

### Food choice identities: factors shaping food choice decisions

Sobal and Bisogni [18] have addressed a number of these choice concepts in their theoretical paper concerning food choice decisions. They characterize food choice decisions as “multi-faceted, situational, dynamic and complex”, and argue that these attributes may be applied to almost any behavioral choice decision. They contrast the social behavior perspective, that food decisions are rational and designed to maximize benefits, against two other theoretical perspectives. The social facts perspective posits that “social institutions and other environments” shape or constrain food choices and the social definition perspective assumes that food choice is an active process whereby individuals “interactively interpret options” in formulating their choices. They highlight the incompatibility of these theoretical frameworks, and introduce the concept of food choice trajectories and transitions, food choice scripts that offer “best-fit solutions”, and food choice behavior that is measured against some external ideal, and thereby also shapes health identity.

Our current discussion advances this concept of choice to suggest a different type of dynamic process, including the assignation of temporal, circumstantial, momentary and relative weight to the different variables inclusive of health identity that figure in each health behavior-making decision. More than choices per se, choice set is a unifying construct, that is comprised of variable, but dialogic and discursive relationships between the individual and their social and policy environments, and the choices in front of them.

This has been illustrated in the recent study by Mulvaney-Day et al. [19], in which the issues of health agency and food choice among fast food restaurant employees, were examined. What makes this study relevant is that the sample was comprised of persons with a similar demographic profile and within a similar setting—students who had worked for a minimum of 6 months in fast food— but who differed with respect to their food choices. They examined two categories of variables, environmental influences and internal psychological factors, both which act upon health agency, and showed how the specific inter-dependence of these categories lead to very different choices amongst individuals.

“Whereas everyone’s food choices were constrained by external factors like time, availability and cost, there was a range in how respondents managed the interaction between these external constraints and their food beliefs, desires and emotions that influenced food choices….some…appeared able to put into practice food preferences and beliefs that were in accord with their professed views of normal eating…others expressed lack of control… and diminished abilities to identify available food options that they thought were healthy.”

This study highlights “the interaction between environmental context and individual health agency” and the ethical dilemma, wherein the environmental context among vulnerable populations generally limits the options that their health agency can act upon. Choice set is the relevant and weighted social, environmental and policy variables that are at an individual’s disposal to be able to make informed health-behavior decisions. These factors or variables consist of both mutable and immutable, affective and rational, intentional and inchoate factors, that relate differently when enacted at a given decision-making moment and in a given context.

These factors reference personal knowledge bases and community interpretations of health values, access health-centered and health-situated social networks, engage relational social and health [19] identities (ethnic, class, community, neighborhood or locale, gender, disability and age-based identities, among others), and occur in specific built, natural and policy environments. Mulvaney-Day and colleagues [19] suggest that public health professionals concerned with healthy eating, for example, must take into account the relative and circumstantial weighting of these various factors, and the extent to which they support or undermine healthy eating behavior, when devising policies and interventions.

### Food consumer behavior and the neighborhood food environment

Another example of the complex weightedness of variables in the choice set may be seen when one considers that in high income countries, the relationship between income and obesity is typically inverse, co-eluting with food insecurity, and that the most disadvantaged are typically the vulnerable, urban poor [20]. In our previous discussion of “vital places”, we focused on the “neighborhood food environment”, disparities in access to healthy foods and the existence of so-called “food deserts”, which have been implicated in poor food choices and the higher prevalence of obesity in these low-income communities [21, 22]. Despite this, there appears to be more to food and food store choice than just proximity. Shannon has shown that low-income families that receive supplemental nutritional benefits are more likely to shop in convenience stores, even when there are supermarkets present, and that there is a net outflow of monies from these social benefits to discount food retailers beyond neighborhood borders [23]. This is confirmed by a recent study from California, in which persons from low-income communities were prepared to drive further to access lower-priced and foods of lower dietary quality, and were three times more likely to be overweight, than their more well-resourced counterparts [21]. For these individuals, supermarket chains offering perceived value propositions were also relationally proximal, despite the fact that they were geographically more distant [24].

Conversely, these results may be contrasted with those recently described in a low- and middle-income country (LMIC) setting such as South Africa, with large income disparities, low levels of car ownership and a lack of equity in the geospatial distribution of supermarkets [25, 26]. Even in lower income communities where supermarkets are located, “consumer segmentation” results in disparities with regard to the stocking of fresh produce, and a disproportionate access to calorie-dense, nutritionally-poor foods [26, 27]. In this regard, the choice set may be both, directly and indirectly, shaped by the environment, the individual and the community.

Clearly, the influences of culture and preference must also be considered, with food purchases being bundled into categories and “triaged” according to whether they are basic food stuffs, treats or rewards. Furthermore, shopping centers are selected on the basis, not only of the value proposition, but something more abstract, such as cleanliness, smells or the way in which individuals are treated as customers [28]. All of these examples suggest that proximity and access are relational and that along with perceived value, preference weighs in strongly in the food choice algorithm, even in low-income settings, to such an extent, that individuals are prepared to travel further just to realize these attributes.

### Food choice within the context of the family

These examples suggest that food choice decisions are evidenced-based, iterative, dialogic and situational, and are informed, consciously or unconsciously, by the environment. Individuals make decisions based on complex interpretations grounded in their own social and health experiences and needs. Slater et al. [29] describe the tension behind the difficult choices Canadian working mothers make, as the primary food providers, between complying with their own healthy food ways values and their identities as “good mothers” and the time constraints that working and pursuing career objectives place upon them.

Family food choice strategies may involve compromising healthier options, while allowing women to construct other meaningful identities. They conclude that the, “confluence of changing workforce dynamics, composition of the food supply, and shifting norms surrounding food provisioning provide an ideal substrate for poor nutritional choices that can lead to obesity” [29]. Therefore, the authors suggest that poor nutrition should *not* be the sole focus of health policy, but that it should rather include the political and social issues concerning flexibility in the workplace and equity in the distribution of domestic work.

**Fig. 1:**
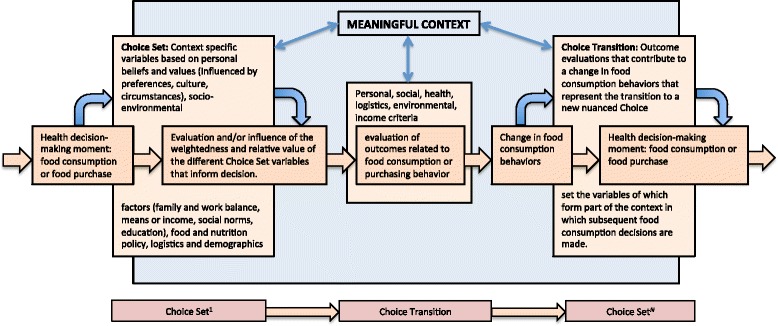
Choice set/choice transition conceptual diagram. The figure shows the relationship of the context-specific variables to food consumption decisions, and subsequent transitions in food consumption behavior based on an evaluation of outcomes that redefine the set of relative variables

These examples introduce choice transition, which represents an essential shift of the choice set based upon the individual’s interpretation of the efficacy of the outcomes of their health-behavior decisions (Fig. 1). It means a new basis for their choice set, that is discursive and iterative and not mechanical or necessarily incremental. Choice transition also posits a different relationship with external forces because the transition is out of a choice set that includes both mutable and immutable variables and on the conscious and unconscious weighting of accumulated evidence such that people can project certain outcomes. This accumulated evidence is based on an analysis of outcomes of previous decisions and the experiences that resulted, the observations of other people making similar decisions, the entitlement or authority of the person or institution providing the information, and the extent to which individuals understand the social context provided by the community that defines health and healthy behavior.

### Relative advantage of these concepts and implications for research, intervention and policies

The concept of choice set recognizes personal and emergent variables that often are as significant as those suggested in a top-down policy context or those that adhere strictly to social and/or environmental determinism. In this regard, people are not destined to simply follow or fail to adhere to public policy recommendations, nor simply respond or not to their immediate environment. In fact, health behavior references that inform decision-making moments may be sources other than public policy interventions, including the individual’s previous social and health experiences, health beliefs and identities, recommendations from credible and entitled authorities, decisional balances and situational opportunities. The mutability and situational character of what are the specific determinant variables in choice sets means that any attempt to reduce it or legislate it from a rigid theoretical or methodological perspective will not recognize the personal investment in the decision-making process, nor the fact that it is grounded in the individual’s social experience.

Hillier, et.al. [30], using a discrete choice experiment, showed how people are agents to their own food purchasing, where invariant factors such as distance to store, price, availability are mediated by how people view the “food landscape”, and that differences in the choice set are based on differentiated and differently valued social, ethnic, gender, and demographic variables suggesting that choice is individual and cannot be simply mapped. They are critical of descriptive approaches that “stop short of modeling the complexities of food shopping behavior and are of limited utility in understanding how households choose food stores, how food store choice influences the foods they purchase, and how food item choice influences health” [30].

Finally, we discuss the construct of choice set applied to health behavior from the perspectives of research and policy design. Choice set does not necessarily lead to “good” choices, but they are informed and perhaps optimised choices, based on the interpretation and appreciation of the information and variables at hand. We argue that to address the growing pandemic of so-called “lifestyle diseases” there must be a comprehensive grounding of the health behavior or decision-making moment with its social and environmental context out of which the prevailing context-specific determinants can be identified.

At the same time, in their model of constrained choice and the impact of certain policy decisions on healthy behavior, Bird and Rieker [31] argue that decision makers at every level share joint responsibility for individual health behavior choices. We suggest that constrained choice resulting from ecological and situational factors require a change in response or a re-evaluation of the weightedness of the different factors that comprise choice set, whereas constrained choice by design, ostensibly directed toward the choice set, requires a change in choice architecture, “drafting social policies with the aim of increasing opportunities for people to pursue better health”.

Public policy interventions tend to be normative, idealizing certain health choices and health profiles [5, 32]. But public policy alone cannot be a predictor of health-behavior choices, in large part because, in spite of the systemic constraints it represents, it cannot guarantee the same outcome at the moment of engagement of the choice set nor, the health decision-making moment. To the contrary, public policy should be directed not so much to a final product nor to establishing a final normative environment to which health identities conform, rather to the offering of tools that enable the individual to better evaluate and weigh the factors that comprise their choice set. Shannon’s example of perpetuating poor food choices under the United States Supplemental Nutrition Assistance Program applies here [23]. Policy cannot accommodate individual choice sets, but can recognise how they work, how they are influenced by meso-level relationships, and how they incorporate the community paradigm that informs and optimizes choice sets, without imposing normative health expectations that disregard local sociocultural values.

Research and public policy interventions are more typically based on categorical bi-variable approaches (environmental/epidemiological, social/epidemiological, and demographic/ epidemiological) with recognition of collateral variables, and only recently have begun to construct more complex integrated approaches toward understanding health behavior. We suggest that this complexity be taken further and that the community-contextualized (meso-level) individual choice set be the minimum unit of analysis to understand health-behavior decision making from a research perspective and instrumental in the design of policy and academic interventions.

## Conclusion

We argue that choice set and choice transition offer more equitable and nuanced concepts of health behavior decision-making to which research and policy interventions may be directed. For example, in terms of food consumption, there will always be choice-constrained conditions, along a continuum representing factors over which the individual has little or no control, to those for which they have greater agency. These range from food store geographies and inventories and food availability, logistical considerations such as transportation, food distribution, the structure of equity in food systems, state and non-government food and nutrition programs, to factors where the individual exercises a greater degree of autonomy, such as sociocultural food ways, family and neighborhood shopping strategies, and personal and family food preferences. At any given food decision-making moment, all or many of these and other factors are present consciously or unconsciously when the individual makes a choice. Policies and interventions should avoid dichotomies of “good and bad” food choices or health behaviors, but focus on those issues that contribute to the weightedness of factors influencing food choice behavior at a given decision-making moment and within a given choice set.
